# Correlation of QRDR mutations and MIC levels in fluoroquinolone-resistant *Staphylococcus aureus* clinical isolates

**DOI:** 10.1128/spectrum.00645-25

**Published:** 2025-10-13

**Authors:** Sohyeong Kim, Xianglan Xuan, Minju Jung, Yujin Park, Soyun Kim, Gaeun Woo, Heechul Park, Sangha Kim, Jiyoung Lee, Min Park, Sunghyun Kim

**Affiliations:** 1 Department of Clinical Laboratory Science, College of Health Sciences, Catholic University of Pusan 65483, Busan, Republic of Korea; 2 Next-Generation Industrial Field-Based Specialist Program for Molecular Diagnostics, Brain Busan 21 Plus Project, Graduate School, Catholic University of Pusan 65483, Busan, Republic of Korea; 3 Department of Forensic Science, Graduate School, Catholic University of Pusan 65483, Busan, Republic of Korea; 4 Department of Clinical Laboratory Science, Division of Natural Sciences, Hyejeon College, Chungcheongnam-do, Republic of Korea; 5 Department of Laboratory Medicine, Konyang University Hospital 68249, Daejeon, Republic of Korea; 6 Department of Research & Development, DreamDX, Busan, Republic of Korea; 7 Department of Biomedical Laboratory Science, Masan University, Changwon, Republic of Korea; Houston Methodist, Houston, Texas, USA

**Keywords:** *Staphylococcus aureus*, antimicrobial resistance (AMR), quinolone resistance-determining region (QRDR), minimum inhibitory concentration (MIC), fluoroquinolone, DNA mutation

## Abstract

Antimicrobial resistance is a global health problem. Among various antibiotic-resistant bacteria, *Staphylococcus aureus*, particularly methicillin-resistant *S. aureus* (MRSA), is a clinically important pathogen responsible for serious infections because of its multidrug resistance (MDR) and association with high mortality rates. The MDR nature of MRSA, including resistance to macrolides, aminoglycosides, fluoroquinolones, and tetracyclines, limits therapeutic choices and poses significant challenges in clinical management. This study aimed to analyze the correlation between mutations in the quinolone resistance-determining region (QRDR) and the minimum inhibitory concentration (MIC) of fluoroquinolone drugs, such as ciprofloxacin and levofloxacin, in MRSA and methicillin-sensitive *S. aureus* (MSSA). A total of 63 *S*. *aureus* clinical strains were isolated from blood samples of sepsis patients. DNA sequence analysis was performed using gDNA extracted from all *S. aureus* clinical isolates to identify mutations in the QRDR of *gyr*A, *gyr*B, *par*C, and *par*E. The MICs of antimicrobials were determined by the broth microdilution method. Among these genes, only mutations in *par*C showed a statistically significant positive correlation with elevated MIC levels, underscoring the primary role of *par*C in mediating resistance in our clinical isolates. Notably, all isolates exhibited a substitution at serine 80 (S80) in *par*C, and those harboring simultaneous substitutions at both S80 and glutamic acid 84 (E84) demonstrated markedly increased MIC values for both drugs. These findings reinforce previously reported associations between dual mutations and high-level fluoroquinolone resistance, while highlighting the distinct contribution of *par*C among the QRDR genes analyzed in this study. Furthermore, we found that the most frequent mutation in the QRDR was the cytosine-to-thymine mutation.

**IMPORTANCE:**

Antimicrobial resistance is a growing global health crisis, making bacterial infections harder to treat. *Staphylococcus aureus*, especially MRSA, is a major concern due to its resistance to multiple antibiotics, including fluoroquinolones like ciprofloxacin and levofloxacin. Our study highlights how specific genetic mutations in the quinolone resistance-determining region (QRDR) influence fluoroquinolone resistance. We found that mutations in the *par*C gene, particularly substitutions at serine 80 (S80) and glutamic acid 84 (E84), significantly increase resistance. Understanding these mutations helps predict antibiotic resistance and may guide more effective treatment strategies. By identifying key genetic changes that drive fluoroquinolone resistance, our research contributes to developing improved diagnostic tools and targeted therapies to combat drug-resistant *S. aureus* infections. This knowledge is crucial for clinicians and researchers working to control the spread of antibiotic-resistant bacteria and improve patient outcomes.

## INTRODUCTION

Antimicrobial resistance (AMR), often referred to as a “silent pandemic,” is one of the global challenges and public health threats humanity faces in the 21st century according to the World Health Organization (WHO). Numerous studies report that by 2050, more deaths from antibiotic-resistant bacterial infections are expected than from cancer (1). In response, global campaigns such as World Antibiotic Awareness Week (WAAW) have been launched to curb the spread of drug-resistant infections. However, antibiotic consumption continues to rise worldwide. For example, the Republic of Korea ranks among the highest in antibiotic use among OECD countries (https://doi.org/10.1787/ae3016b9-en), highlighting the urgency of local and global efforts to address this crisis.

*Staphylococcus aureus* is a Gram-positive bacterium commonly found on the skin and in the nasal passages of healthy individuals (2).

Although often harmless in healthy individuals, *S. aureus* can cause severe infections in immunocompromised or hospitalized patients, including skin abscesses, pneumonia, bloodstream infections, and sepsis, particularly when introduced through wounds or invasive procedures.

Sepsis is a life-threatening condition marked by dysregulated inflammatory responses to infection, potentially leading to multi-organ failure or death (3). According to the US CDC, one in three hospital deaths is related to sepsis, with a growing burden worldwide. Given its high mortality and complication rates, early identification and appropriate antimicrobial treatment are essential (4, 5).

Among the various strains of *S. aureus*, methicillin-resistant *S. aureus* (MRSA) poses a significant therapeutic challenge due to its resistance to beta-lactam antibiotics. The World Health Organization classifies MRSA as a high-priority antimicrobial-resistant pathogen, underscoring its role in severe and difficult-to-treat infections globally.

Methicillin is a penicillin-class antibiotic developed to resist degradation by beta-lactamase enzymes, which inactivate earlier beta-lactam antibiotics such as penicillin by breaking the beta-lactam ring. Like other beta-lactams, methicillin inhibits bacterial cell wall synthesis. Like other penicillin antibiotics, methicillin binds to penicillin-binding proteins (PBPs) to inhibit bacterial cell wall synthesis. However, the *mec*A gene, which encodes PBP2a—a low-affinity PBP that is not affected by beta-lactam antibiotics—could be transferred through the staphylococcal cassette chromosome *mec*, resulting in MRSA (6, 7). MRSA infections are commonly seen in healthcare settings (healthcare-acquired MRSA, or HA-MRSA), often due to invasive medical devices such as catheters, but unfortunately, MRSA is now also spreading to the community (community-acquired MRSA, or CA-MRSA) (8, 9).

In the Republic of Korea, MRSA is considered a risk factor for healthcare-associated infections (HAIs) and is designated and monitored as a legal infectious disease by the Korea Centers for Disease Control and Prevention (KCDC). It is especially prevalent in Asia (8, 10, 11). Furthermore, MRSA strains not only exhibit intrinsic resistance to all β-lactam antibiotics but also frequently acquire resistance to other antibiotic classes, including macrolides, aminoglycosides, fluoroquinolones, and tetracyclines, rendering treatment options increasingly limited. Therefore, the therapeutic limitations for MDR *S. aureus*, its resistance to antibiotics, proper management of antibiotic use, and the prevention of infection must be addressed.

One of the antibiotic treatments for MRSA is vancomycin. However, vancomycin currently presents a significant challenge for the healthcare system because its use is limited due to its high cost, difficulty in managing intravenous (IV) administration, numerous serious side effects, and increasing reports of vancomycin-resistant bacteria worldwide (12).

Fluoroquinolones are a class of quinolone antibiotics that have a bactericidal effect by inhibiting the activity of DNA gyrase and topoisomerase IV, enzymes involved in DNA synthesis and replication. These drugs are among the most prescribed antimicrobial agents worldwide (13). Ciprofloxacin and levofloxacin are the most commonly used fluoroquinolones (14). Ciprofloxacin, a second-generation fluoroquinolone, has been designated by the WHO as an essential drug and is widely prescribed. Levofloxacin, a third-generation fluoroquinolone, was developed with enhanced activity against gram-positive bacteria (13, 15). Recently, it has been reported that MRSA resistant to fluoroquinolones is increasing and is difficult to treat (16). In comparison to ciprofloxacin-resistant strains, MRSA showed the second-highest resistance percentage (90%) after vancomycin-resistant enterococci (17). Furthermore, ciprofloxacin was the third most commonly identified resistant drug in *S. aureus* isolates analyzed by the KCDC between 2016 and 2022, with a resistance rate of 31.6% (https://nih.go.kr/nohas/en/statistics/selectARStatisticsMainTab.do?systemName=Kor_GLASS). Additionally, studies have suggested that ciprofloxacin should no longer be used as a treatment for MRSA infections and should only be used in limited cases (12).

The mechanisms by which fluoroquinolone resistance is acquired can be divided into three categories: chromosomal mutations in drug target enzymes, efflux pump overexpression, and plasmid-mediated resistance gene acquisition. Among these, resistance due to chromosomal mutations in target enzymes is the most common mechanism (13). These antibiotic resistance mechanisms arise when mutations occur at the subunit sites of the target enzymes, DNA gyrase (which regulates DNA supercoiling), and topoisomerase IV (which relaxes supercoiled DNA). The subunit sites, including *gyr*A and *gyr*B of DNA gyrase, and *par*C and *par*E of topoisomerase IV, are collectively referred to as the quinolone resistance-determining region (QRDR) (18).

Therefore, the present study analyzed DNA mutations and amino acid substitutions in the subunit sites of the QRDR and determined the minimum inhibitory concentration (MIC) levels by conducting a broth microdilution method for ciprofloxacin and levofloxacin in clinical isolates of fluoroquinolone-resistant *S. aureus* isolated from patients with sepsis. Based on these results, statistical analysis was performed to investigate the correlation between the degree of mutation in the QRDR and the MIC levels in order to identify the sites associated with resistance.

## MATERIALS AND METHODS

### Clinical isolates

In the present study, a total of 63 *S*. *aureus* clinical isolates from blood culture samples were collected from patients with sepsis admitted to Konyang University Hospital, a general hospital with a capacity of 850 beds located in Daejeon, the Republic of Korea, between August 2015 and June 2018. The samples were provided by the Diagnostic Microbiological Laboratory of the Department of Laboratory Medicine. *S. aureus* clinical strains were single clones obtained from one or more pairs of positive blood culture bottles containing pediatric or adult samples, without including duplicate strains. For analysis, the strains were divided into the categories of MRSA and methicillin-sensitive *S. aureus* (MSSA) based on the antimicrobial (oxacillin) susceptibility test results.

### Blood culture

Blood cultures were performed at the affiliated hospital’s Diagnostic Microbiology Laboratory using standard protocols. Isolates identified as *S. aureus* were collected for further analysis in the present study.

### Bacterial identification using MALDI-TOF mass spectrometer

All strains isolated and cultured as single colonies from the blood cultures were subjected to bacterial species identification using a Microflex MALDI Biotyper (Bruker Daltonics, Bremen, Germany). MALDI Biotyper RTC software version 3.1 (Bruker Daltonics) was used to analyze the bacterial species identification results. For the MALDI-TOF mass spectrometry (MS) analysis, fresh single colonies cultured for 16–18 h were smeared directly onto an MSP 96 Target Polished Steel BC Microscout Target Plate (Bruker Daltonics) using a sterilized wooden applicator. After the bacteria had dried, 1 µL of reagent solution containing α-cyano-4-hydroxycinnamic acid saturated with a matrix solution (50% acetonitrile, 2.5% trifluoroacetic acid) was added. After the reagent solution had dried completely at room temperature, the plate was mounted on the Microflex MALDI Biotyper equipment for analysis. The operational definitions were as follows: if the cut-off score set by the equipment manufacturer was 2.0 or greater, species identification was considered valid; if the cut-off score was 1.7 or greater but less than 2.0, bacterial genus identification was possible; and if the cutoff score was less than 1.7, the result was judged as unreliable.

### Antimicrobial susceptibility test

Single colonies obtained after primary culture of the blood samples were adjusted to McFarland 0.5 using the BBL PROMPT Inoculation System (Beckman Coulter, West Sacramento, CA, USA), applied to the Positive MIC 28 Panel with a Renok Inoculator, and then tested using the MicroScan WalkAway 96 Plus System (Beckman Coulter). The MIC level was classified as susceptible, intermediate, or resistant according to the CLSI guidelines, CLSI M100 S30. Through this process, ciprofloxacin resistance was confirmed.

### Genomic DNA extraction

A total of 63 *S*. *aureus* genomic DNA (gDNA) samples were extracted from the single colonies isolated from the blood-derived samples for molecular genetic characterization. Single colonies of the cultured bacteria were collected using a platinum loop. For the washing step, 1,000 µL of distilled water (DW) was added, mixed well, and centrifuged at 12,000 × *g* for 2 min. After removing the supernatant, only the remaining cell pellets were stored in a deep freezer for 5 min. The pellets were then incubated at 100°C for 10 min after adding 200 µL of 5% Chelex-100 Resin (Bio-Rad, Hercules, CA, USA). Following incubation, the sample was centrifuged at 12,000 × *g* for 2 min, and only the supernatant was retained. The supernatant served as the template DNA for the PCR. After confirming the purity and concentration using a NanoDrop 2000 Spectrophotometer (Thermo Fisher Scientific, Waltham, MA, USA), the extracted gDNA was stored at –20°C until further analysis.

### *mec*A gene detection using qPCR SYBR Green assay

To detect the *mec*A gene, the quantitative polymerase chain reaction (qPCR) SYBR Green assay was performed as follows: 10 µL of THUNDERBIRD SYBR qPCR Mix (Toyobo, Osaka, Japan), 10 pmol of each forward and reverse primer, 3 µL of extracted gDNA as template DNA, and 5 µL of sterile DW were added to prepare a 20 µL reaction mixture. The qPCR SYBR Green assay was conducted using the QuantStudio 7 Flex Real-Time PCR Instrument (Thermo Fisher Scientific). The nucleotide sequences of the primer pairs were designed based on existing research data (Table 1) (19). The qPCR conditions for detecting the *mec*A gene were as follows: pre-denaturation at 94°C for 5 min, 30 cycles of denaturation at 94°C for 30 s, primer annealing at 50°C for 30 s, and extension at 72°C for 30 s, followed by a final extension at 72°C for 10 min. After the amplification process was completed, a melting curve was derived by increasing the temperature of the amplification product in 0.5°C increments, from 55°C to 95°C. The specificity of the primer pair used was then determined. The qPCR result was considered valid only when the cycle threshold value of the amplification curve derived from the qPCR was less than 30.

**TABLE 1:** Oligonucleotide primer pairs used for target gene detection in this study

Target genes	Primer sequence (5′ to 3′)		Amplicon size (bp)	References
*mec*A	Forward GTG AAG ATA TAC CAA GTG ATT		147	(19)
	Reverse ATG CGC TAT AGA TTG AAA GGA T			
*gyr*A	Forward CAG GAC CTT CAA TAT CCT CC		574	(10)
	Reverse GCG ATG AGT GTT ATC GTT GCT			
*gyr*B	Forward CGA TTT TGT GAT ATC TTG CTT TCG		291	
	Reverse CAG CGT TAG ATG TAG CAA GC			
*par*C	Forward GTT GGA AAA TCG GAC CTT		664	
	Reverse GAT GAG GAG GAA ATC TAG			
*par*E	Forward CAT CAG TCA TAA TAA TTA CAC		405	
	Reverse GAC AAT TGT CTA AAT CAC TTG TG			

### QRDR gene detection analysis

**Fig 1:**
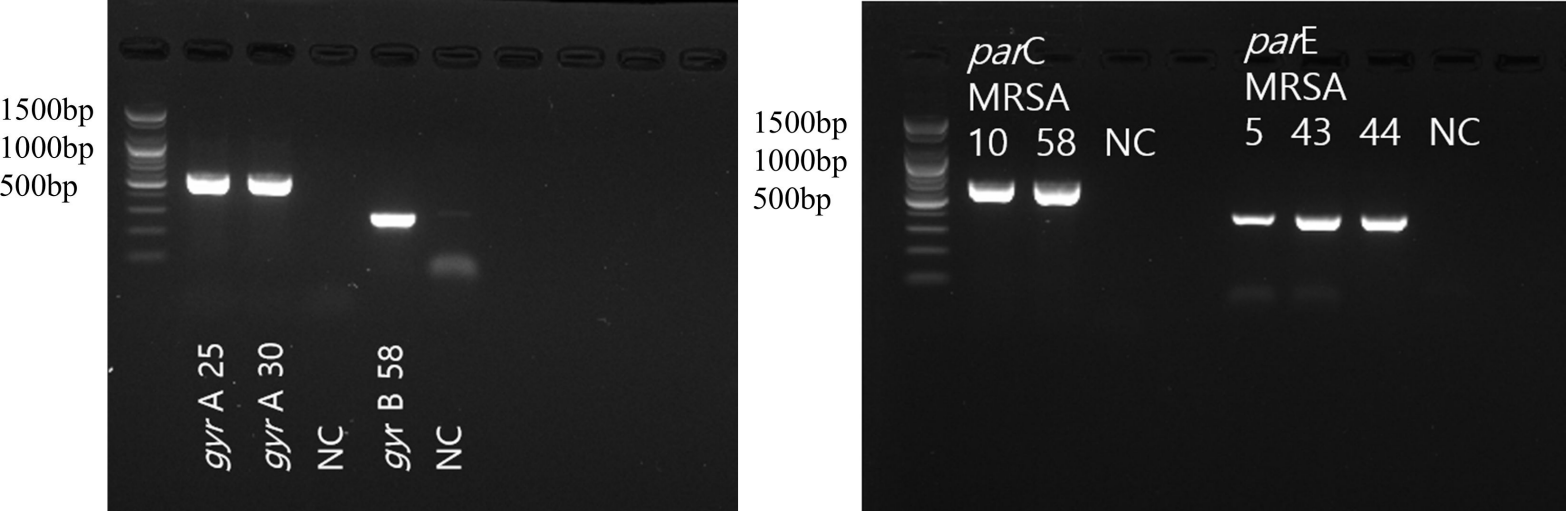
Confirmation of amplified *gyr*A, *gyr*B, *par*C, and *par*E genes using DNA electrophoresis. Amplicon size (bp): *gyr*A = 574, *gyr*B = 291, *par*C = 664, and *par*E = 405. NC: negative control.

In the present study, the *gyr*A, *gyr*B, *par*C, and *par*E genes in the QRDR were detected by conventional PCR in *S. aureus* isolates to identify mutations in the DNA gyrase and topoisomerase IV enzymes. The nucleotide sequences of the primer pairs were designed based on existing research data (Table 1) (10). Each primer was diluted to 10 pmol. The PCR mixtures were prepared in a total volume of 20 µL. Each reaction contained 3 µL of template DNA, 1 µL of each primer, 10 µL of Prime Taq Premix (2×) (GeNet Bio, Daejeon, Republic of Korea), and 5 µL of ultra pure water. The PCR amplification conditions were as follows: pre-denaturation for 5 min at 95°C, followed by 35 cycles of denaturation at 95°C for 30 s, annealing at 50°C for 30 s, and extension at 72°C for 30 s, with a final extension at 72°C for 5 min. The PCR was performed using a SimpliAmp Thermal Cycler (Thermo Fisher Scientific). PCR products were then confirmed by DNA electrophoresis on 2.0% agarose gel (Fig. 1).

### DNA sequence analysis of QRDR with gDNA samples and analysis of mutation

**Fig 2:**
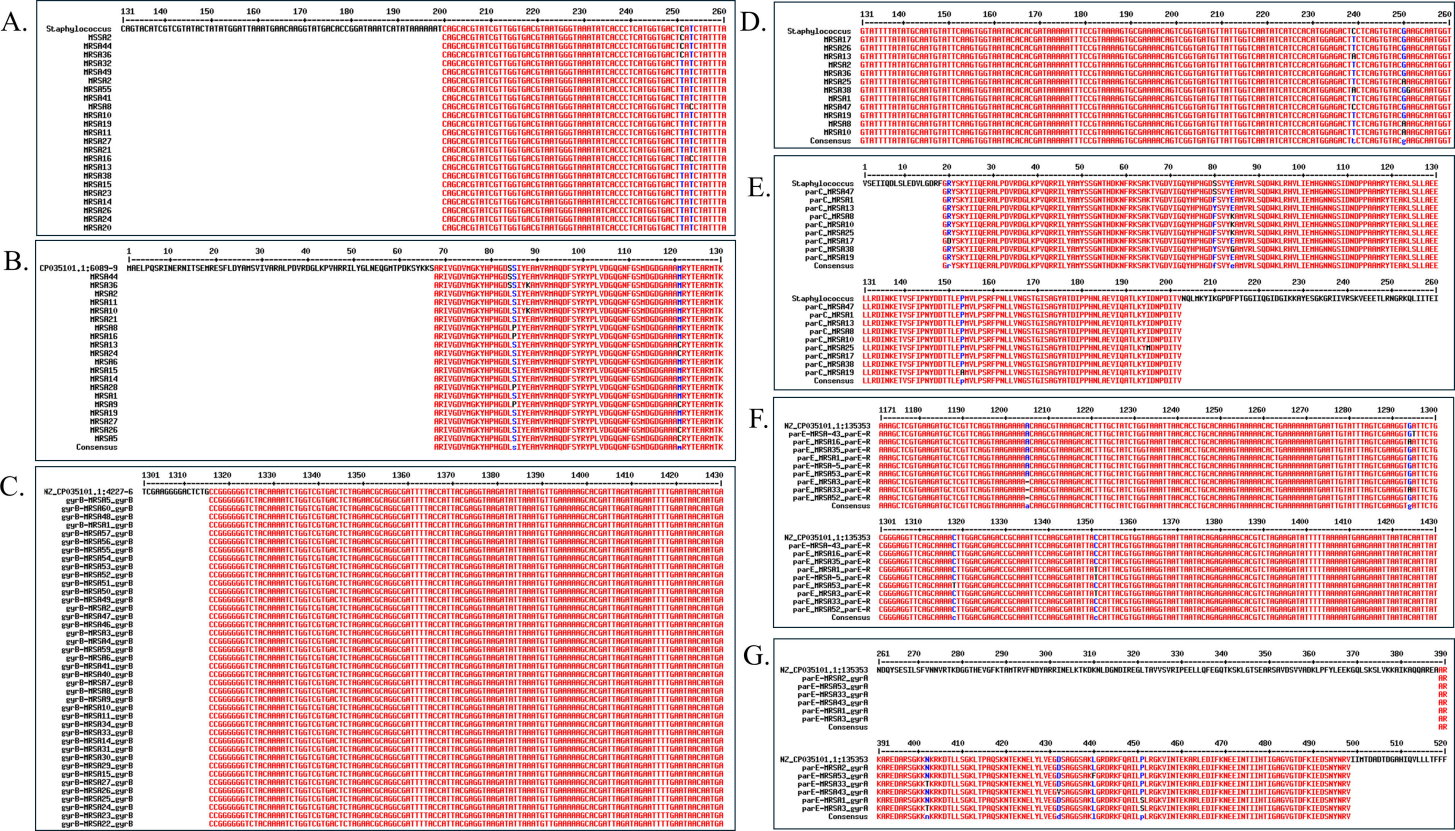
Analysis of DNA and amino acid mutations compared to *S. aureus* ATCC 12600 using Multalin. (**A**) *gyr*A gene nucleotide sequence, (**B**) GyrA amino acid sequence, (**C**) *gyr*B gene nucleotide sequence, (**D**) *par*C gene nucleotide sequence, (**E**) ParC amino acid sequence, (**F**) *par*E gene nucleotide sequence, (**G**) ParE amino acid sequence.

DNA sequence analysis of *gyr*A, *gyr*B, *par*C, and *par*E in the QRDR was conducted with gDNA samples obtained from the Macrogen Online Sequencing Order System (Macrogen, Seoul, Republic of Korea). To analyze the DNA mutations, the obtained DNA sequence was compared with *S. aureus* ATCC 12600 using the Multalin interface (http://multalin.toulouse.inra.fr/multalin/) (20). An example of the alignment method used to detect QRDR mutations is shown in Fig. 2. Additionally, amino acid substitution analysis was performed on the protein sequence. The nucleotide sequence was translated into an amino acid sequence using the Translate Tool provided by Expasy (https://web.expasy.org/translate/).

### Determination of MIC levels of fluoroquinolone antibiotics using the broth microdilution method

**Fig 3:**
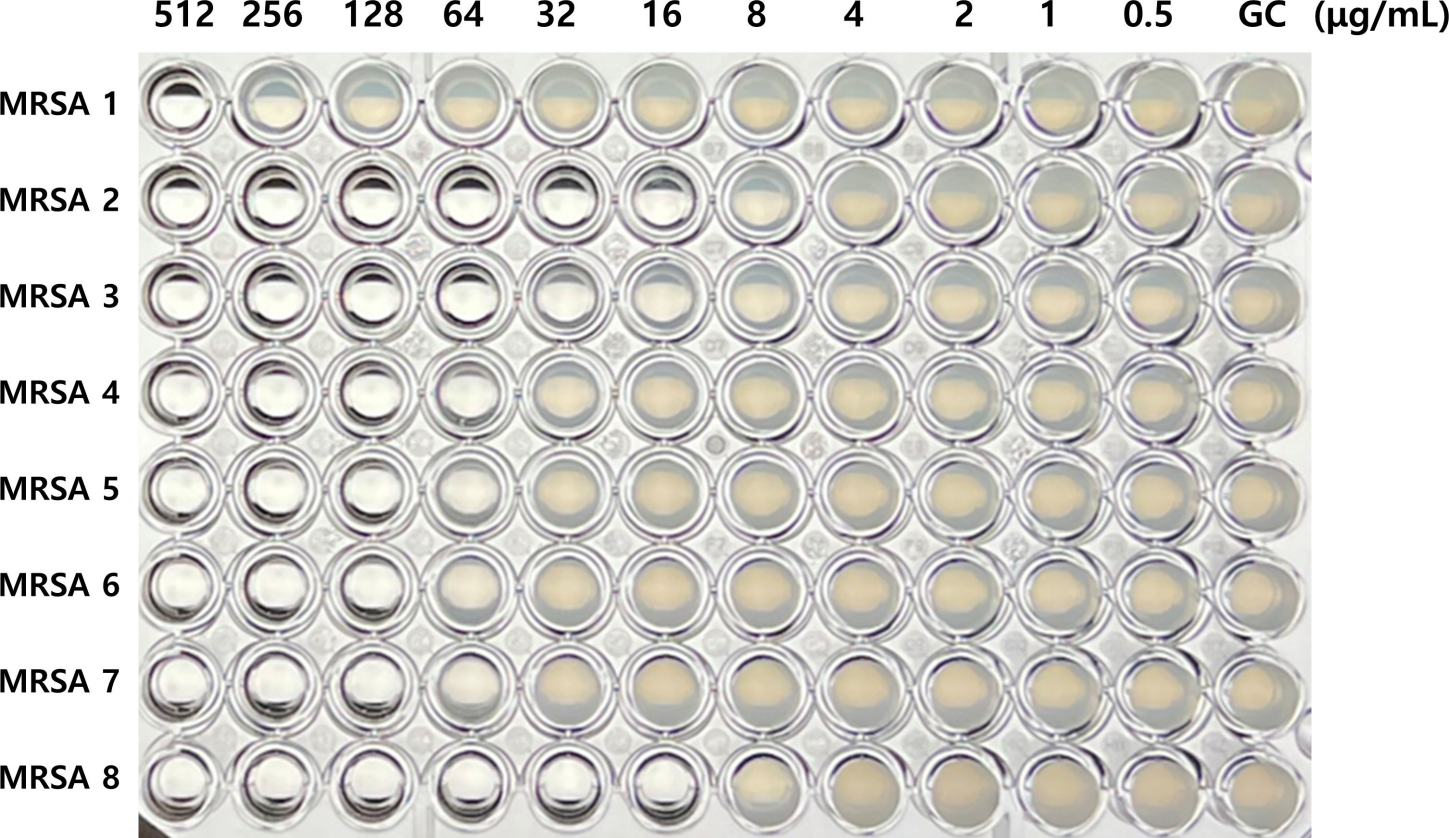
Detection of minimum inhibitory concentration (MIC) levels of fluoroquinolone antibiotics using the broth microdilution method. GC: growth control.

The MIC levels for ciprofloxacin and levofloxacin were determined by the broth microdilution method. For preculture, a total of 63 *S*. *aureus* isolates were inoculated in Mueller–Hinton broth and incubated at 37°C for 20 h. After incubation, the cultures were adjusted to 0.5 McFarland (1.5 × 10^8^ CFU/mL), followed by dilution to 1.0 × 10^6^ CFU/mL. The concentrations of both antibiotics were prepared ranging from 512.0 to 0.5 µg/mL using a twofold serial dilution. A 1.0 × 10^6^ CFU/mL dilution of bacteria was dispensed into a 96-well plate containing each concentration of antibiotics (100 µL per well) and incubated at 37°C for 20 h (Fig. 3). The absorbance at 595 nm was measured using a microplate reader (iMARK Microplate Reader, Bio-Rad) to assist in determining the MIC level in cases where visual inspection was ambiguous. The MIC was defined as the concentration at which the absorbance was reduced by more than half compared to the previous concentration.

### Statistical analysis

The creation of graphs and the correlation analysis between MIC levels and the degree of DNA mutations and amino acid substitutions in the QRDR were conducted using GraphPad Prism 8.4.3 software (GraphPad Software, San Diego, CA, USA). Additionally, a linear regression analysis was performed on the correlation graphs. Comparison of ciprofloxacin and levofloxacin MIC levels according to mutation site groups was analyzed using Tukey’s multiple comparisons test. *P*-values < 0.05 were considered statistically significant.

## RESULTS

### Isolation of *S. aureus* clinical isolates

A total of 141 *S*. *aureus* isolates were identified by MALDI-TOF MS analysis using a single colony isolated from blood cultures. Of these, 63 *S*. *aureus* strains were confirmed to be resistant to ciprofloxacin through antimicrobial susceptibility testing of the 141 blood culture samples. These 63 strains were further classified into 60 MRSA and 3 MSSA by the *mec*A qPCR SYBR Green assay (Table 2).

**TABLE 2:** Total number of clinical samples used in the present study

Clinical samples (*n* = 63)	Parameter	Detail	*n* (%)
MRSA (*n* = 60)	Sex	Male	33 (55)
		Female	27 (45)
	Age	0–9	4 (6)
		10–19	0 (0)
		20–29	0 (0)
		30–39	2 (3)
		40–49	3 (5)
		50–59	4 (6)
		60–69	13 (21)
		70–79	25 (41)
		80–09	9 (15)
	Neutrophil count	>40%–<70%	48 (80)
		<40% or >70%	12 (20)
	CRP level	<0.5 mg/dL	57 (95)
		>0.5 mg/dL	3 (5)
MSSA (*n* = 3)	Sex	Male	1 (33)
		Female	2 (66)
	Age	0–9	0 (0)
		10–19	0 (0)
		20–29	0 (0)
		30–39	0 (0)
		40–49	1 (33)
		50–59	0 (0)
		60–69	0 (0)
		70–79	2 (66)
		80–09	0 (0)
	Neutrophil count	>40%–<70%	1 (33)
		<40% or >70%	2 (66)
	CRP level	<0.5 mg/dL	3 (100)
		>0.5 mg/dL	0 (0)

### QRDR sequencing and mutation analysis

**Fig 4:**
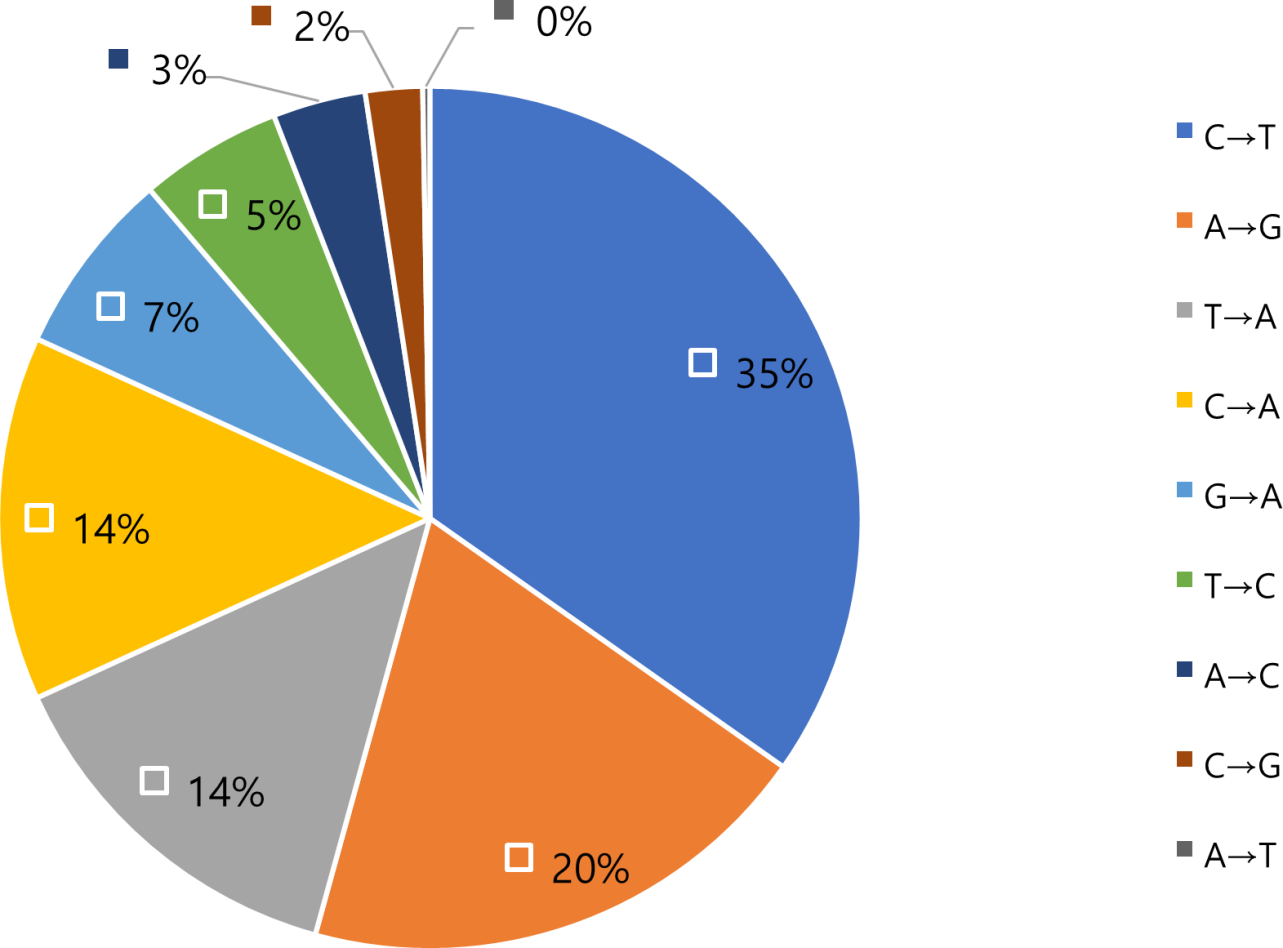
Count of DNA base changes in QRDR sites of fluoroquinolone-resistant *S. aureus*.

The QRDR sequencing analysis of a total of 63 *S*. *aureus* strains isolated from patients with sepsis was conducted and compared with *S. aureus* ATCC 12600 to identify DNA mutations. The results of the mutation analysis of gDNA and amino acids in the QRDR, including *gyr*A, *gyr*B, *par*C, and *par*E genes, are shown in Tables 3 to 6. No mutations were observed in the *gyr*B gene; therefore, this gene was excluded from the results. Based on the analysis results, the frequency of gDNA mutations and amino acid substitutions was organized in ascending order (Tables 7 and 8). An asteriskindicates a stop codon, and “ND” indicates that mutations were not detected. In the *gyr*A gene, 94% of mutations resulted in the conversion of cytosine (C) to thymine (T) at position 251. In the *par*C gene, 84% of mutations resulted in the conversion of C to T at position 239, and in the *par*E gene, the C to T mutation at position 1,351 was observed in 25% of cases. More than 50% of the DNA mutations were absent in the *par*E gene. This confirms that the most frequent DNA mutation in the QRDR region is the C to T mutation (Fig. 4). Furthermore, the highest frequency of amino acid substitution in the QRDR was found in the GyrA subunit, where 59 (94%) samples exhibited S84L substitution, and in the ParC subunit, 53 (84%) samples exhibited S80F substitution (Table 8). In contrast, no substitution was found in 41 (65%) samples in the ParE subunit. The results of the MIC test for ciprofloxacin and levofloxacin using the broth microdilution method are also shown in Tables 9 and 10.

**TABLE 3:** DNA changes detected in the QRDR, including *gyr*A, *gyr*B, *par*C, and *par*E genes in fluoroquinolone-resistant MRSA clinical isolates^*a*^

Strain Gene	1	2	3	4	5	6	7	8	9	10	11	12	13	14	15
*gyr*A	C251T	C251T	C251T	C251T	C251T	C251T	C251T	C251T	C251T	C251T	C251T	C251T	C251T	C251T	C251T
	361delA	A501G	A501G	A501G	361delA	391delA	A501G	T253C	T253C	G262A	G451A	T253C	361delA	T500C	A501G
	A501G	T513A	T513A	T513A	A418G	A501G	T513A	A501G	361delA	A501G	A501G	A501G		C525G	T513A
	T513A	C525A	C525A	C525A	451delG	T513A	C525A	T513A	451delG	T513A	T513A	T513A		543delG	C525A
	C525G				A501G	C525A		C525A	A501G	C525A	C525G	C525A			543delG
	563delT				T513A				T513A		A570C				
					C525A				C525G						
					T563A										
*gyr*B	ND	ND	ND	ND	ND	ND	ND	ND	ND	ND	ND	ND	ND	ND	ND
*par*C	ND	C239T	C239T	C239T	C239T	C239T	C239T	C239T	C239T	C239T	C239T	C239T	C239A	C239A	C239T
								G250A	G250A	G250A		G250A			
										608delA					
										612delA					
*par*E	ND	ND	1205delA	ND	C1351T	ND	ND	ND	ND	ND	C1351T	ND	ND	ND	ND
			C1351T												

Strain Gene	16	17	18	19	20	21	22	23	24	25	26	27	28	29	30
*gyr*A	C251T	C251T	C251T	C251T	C251T	C251T	C251T	C251T	C251T	C251T	C251T	C251T	C251T	C251T	C251T
	T253C	T253C	T253C	A501G	361delA	G451A	T253C	G451A	361delA	T253C	361delA	A418G	T253C	A501G	A501G
	A501G	A501G	A501G	T513A	391delA	A501G	A501G	A501G	391delA	A501G	391delA	T500C	391delA	T513A	T513A
	T513A	T513A	T513A	C525G	G451A	T513A	T513A	T513A	A501G	T513A	A418G	A501G	A501G	C525A	C525A
	C525A	C525A	C525A	T563A	A501G	C525A	C525A	C525A	T513A	C525A	A501G	C525G	T513A	543delG	
	527insG				T513A	527insG		543delG	C525A		C525G	T563A	C525A		
					C525A						T563A	A570C	543delG		
											A570C				
*gyr*B	ND	ND	ND	ND	ND	ND	ND	ND	ND	ND	ND	ND	ND	ND	ND
*par*C	C239T	57delA	C239T	C239T	C239T	C239T	C239T	C239T	C239T	C239T	C239T	C239T	C239T	C239T	C239T
		C239T		C457G						G250A	A456G	A456G			
										585delT	A475C	A475C			
*par*E	G1294A	G1294A	G1294A	C1351T	ND	1205delA	G1294A	C1351T	C1351T	ND	ND	ND	G1294A	C1351T	C1351T
						C1351T									

Strain Gene	31	32	33	34	35	36	37	38	39	40	41	42	43	44	45
*gyr*A	C251T	C251T	C251T	C251T	C251T	G262A	C251T	C251T	C251T	C251T	C251T	C251T	C251T	A501G	C251T
	A501G	A501G	T253C	A501G	T253C	A501G	A501G	361delA	A501G	A501G	A501G	A501G	G262A		A501G
	T513A		A501G	T513A	A501G		T513A	A501G	T513A	T513A	T513A	T513A	A501G		T513A
	C525A		T513A	C525A	T513A		C525A	T513A	C525A	C525A	C525A	C525A	T513A		C525A
			C525A		C525A			C525A			A570C		C525A		543delG
*gyr*B	ND	ND	ND	ND	ND	ND	ND	ND	ND	ND	ND	ND	ND	ND	ND
*par*C	C239T	C239T	C239T	C239T	C239T	C239T	C239T	C239A	C239T	C239T	C239T	C239T	C239T	C239T	C239A
		A456G			G250A	C397T	G250A	A251G						A456G	A251G
		A475C				A456G		T376C						A475C	T376C
						A475C									
*par*E	1205delA	ND	1205delA	C1351T	G1459A	ND	ND	ND	C1351T	C1351T	ND	ND	A1295T	ND	ND
	C1351T		G1294A												

Strain Gene	46	47	48	49	50	51	52	53	54	55	56	57	58	59	60
*gyr*A	C251T	A501G	C251T	C251T	C251T	C251T	C251T	C251T	C251T	C251T	C251T	C251T	C251T	C251T	C251T
	T253C		A501G		A501G	A501G			A501G	A501G	T253C	A501G	A501G	A501G	A501G
	A501G				T513A	T513A				T513A	A501G	T513A	T513A	T513A	
	T513A				C525A	C525A					T513A	C525A	C525A	C525A	
	C525A										C525A				
*gyr*B	ND	ND	ND	ND	ND	ND	ND	ND	ND	ND	ND	ND	ND	ND	ND
*par*C	C239T	A456G	C239T	C239A	C239T	C239T	C239T	C239A	C239T	C239A	C239T	C239A	C239T	C239T	C239T
		A475C	A456G						A456G	A251G		A251G		G250A	A456G
			A475C						A475C	T376C		T376C			A475C
*par*E	G1294A	ND	ND	ND	C1351T	ND	1205delA	C1318T	ND	ND	1205delA	ND	C1351T	ND	1205delA
											G1294A				

^*a*^ ND, not detected.

**TABLE 4:** DNA changes detected in the QRDR, including *gyr*A, *gyr*B, *par*C, and *par*E genes in fluoroquinolone-resistant MSSA clinical isolates

Gene	Strain 1	Strain 2	Strain 3
*gyr*A	C251T A501G T513A C525A	ND^*a*^	C251T T253C A501G T513A C525A
*gyr*B	ND	ND	ND
*par*C	C239T	ND	C239T
*par*E	G1294A	ND	G1294A

^*a*^ ND, not detected.

**TABLE 5:** Amino acid substitutions detected in the QRDR, including GyrA, GyrB, ParC, and ParE in fluoroquinolone-resistant MRSA clinical isolates^*a*^

Strain	1	2	3	4	5	6	7	8	9	10	11	12	13	14	15
Region															
GyrA	S84L	S84L	S84L	S84L	S84L	S84L	S84L	S84L	S84L	S84L	S84L	S84L	S84L	S84L	S84L
	M121C				M121C	I131S		S85P	S85P	E88K	I175M	S85P	M121C	L167S	N182I
	I175M				D151*				M121C					I175M	
	L188Q				L188*				D151M					N182I	
									I175M						
GyrB	ND^*b*^	ND	ND	ND	ND	ND	ND	ND	ND	ND	ND	ND	ND	ND	ND
ParC	S80F	S80F	S80F	S80F	S80F	S80F	S80F	S80F	S80F	S80F	S80F	S80F	S80Y	S80Y	S80F
								E84K	E84K	E84K		E84K			
										N203I					
										Q204H					
ParE	P451S	ND	N402T	ND	P451S	ND	ND	ND	ND	ND	P451S	ND	ND	ND	ND
			P451S												

Strain Region	16	17	18	19	20	21	22	23	24	25	26	27	28	29	30
GyrA	S84L	S84L	S84L	S84L	S84L	S84L	S84L	S84L	S84L	S84L	S84L	S84L	S84L	S84L	S84L
	S85P	S85P	S85P	I175M	M121C	A176G	S85P	N182I	M121C	S85P	M121C	L167S	S85P	N182I	
	A176G			L188*	I131S				I131S		I131S	I175M	I131S		
											I175M	L188*	N182I		
											L188*				
											M210E				
GyrB	ND	ND	ND	ND	ND	ND	ND	ND	ND	ND	ND	ND	ND	ND	ND
ParC	S80F	R20D	S80F	S80F	S80F	S80F	S80F	S80F	S80F	S80F	S80F	S80F	S80F	S80F	S80F
		S80F		P153A						E84K					
										I195M					
ParE	ND	ND	ND	P451S	ND	N402T	ND	P451S	P451S	ND	ND	ND	ND	P451S	P451S
						P451S									

Strain Region	31	32	33	34	35	36	37	38	39	40	41	42	43	44	45
GyrA	S84L	S84L	S84L	S84L	S84L	E88K	S84L	S84L	S84L	S84L	S84L	S84L	S84L	ND	S84L
			S85P		S85P			M121C					E88K		N182I
GyrB	ND	ND	ND	ND	ND	ND	ND	ND	ND	ND	ND	ND	ND	ND	ND
ParC	S80F	S80F	S80F	S80F	S80F	S80F	S80F	S80Y	S80F	S80F	S80F	S80F	S80F	S80F	S80Y
					E84K		E84K	E84G							E84G
ParE	N402T	ND	N402T	P451S	ND	ND	ND	ND	P451S	P451S	ND	ND	D432V	ND	ND
	P451S														

Strain Region	46	47	48	49	50	51	52	53	54	55	56	57	58	59	60
GyrA	S84L	ND	S84L	S84L	S84L	S84L	S84L	S84L	S84L	S84L	S84L	S84L	S84L	S84L	S84L
	S85P										S85P				
GyrB	ND	ND	ND	ND	ND	ND	ND	ND	ND	ND	ND	ND	ND	ND	ND
ParC	S80F	ND	S80F	S80Y	S80F	S80F	S80F	S80Y	S80F	S80Y	S80F	S80Y	S80F	S80F	S80F
										E84G		E84G		E84K	
ParE	ND	ND	ND	ND	P451S	ND	N402T	L440F	ND	ND	N402T	ND	P451S	ND	N402T

^*a*^ "*” indicates stop codon.

^*b*^ ND, not detected.

**TABLE 6:** Amino acid substitutions detected in the QRDR, including GyrA, GyrB, ParC, and ParE in fluoroquinolone-resistant MSSA clinical isolates

Region	Strain 1	Strain 2	Strain 3
GyrA	S84L	ND^*a*^	S84L S85P
GyrB	ND	ND	ND
ParC	S80F	ND	S80F
ParE	ND	ND	ND

^*a*^ ND, not detected.

**TABLE 7:** Frequency of DNA changes detected in the QRDR, including *gyr*A, *gyr*B, *par*C, and *par*E genes in fluoroquinolone-resistant MRSA and MSSA clinical isolates from blood culture^*b*^

*gyr*A				*par*C				*par*E			
DNA mutations	Total *n* (%)	MRSA *n* (%)	MSSA *n* (%)	DNA mutations	Total *n* (%)	MRSA *n* (%)	MSSA *n* (%)	DNA mutations	Total *n* (%)	MRSA *n* (%)	MSSA *n* (%)
C251T	59 (94)	57 (95)	2 (67)	C239T	53 (84)	51 (85)	2 (67)	C1351T	16 (25)	16 (27)	0 (0)
A501G	57 (90)	55 (92)	2 (67)	A456G	9 (14)	9 (15)	0 (0)	G1294A	10 (16)	8 (13)	2 (67)
T513A	48 (76)	46 (77)	2 (67)	A475C	9 (14)	9 (15)	0 (0)	1205delA	7 (11)	7 (12)	0 (0)
C525A	43 (68)	41 (68)	2 (67)	C239A	8 (13)	8 (13)	0 (0)	A1295T	1 (2)	1 (2)	0 (0)
T253C	14 (22)	13 (22)	1 (33)	G250A	8 (13)	8 (13)	0 (0)	C1318T	1 (2)	1 (2)	0 (0)
361delA	8 (13)	8 (13)	0 (0)	A251G	4 (6)	4 (7)	0 (0)	G1459A	1 (2)	1 (2)	0 (0)
C525G	7 (11)	7 (12)	0 (0)	T376C	4 (6)	4 (7)	0 (0)	ND^*a*^	33 (52)	32 (53)	1 (33)
543delG	6 (10)	6 (10)	0 (0)	57delA	1(2)	1 (2)	0 (0)				
391delA	5 (8)	5 (8)	0 (0)	585delT	1 (2)	1 (2)	0 (0)				
A570C	4 (6)	4 (7)	0 (0)	608delA	1 (2)	1 (2)	0 (0)				
G451A	4 (6)	4 (7)	0 (0)	612delA	1 (2)	1 (2)	0 (0)				
T563A	4 (6)	4 (7)	0 (0)	C397T	1 (2)	1 (2)	0 (0)				
A418G	3 (5)	3 (5)	0 (0)	C457G	1 (2)	1 (2)	0 (0)				
G262A	3 (5)	3 (5)	0 (0)	ND	2 (3)	1 (2)	1 (33)				
451delG	2 (3)	2 (3)	0 (0)								
527insG	2 (3)	2 (3)	0 (0)								
T500C	2 (3)	2 (3)	0 (0)								
563delT	1 (2)	1 (2)	0 (0)								
ND	1 (2)	0 (0)	1 (33)								

^*a*^ ND, not detected.

^*b*^ Empty cells indicate that the corresponding DNA mutation was not observed for that gene, as mutation types vary among *gyr*A, *par*C, and *par*E.

**TABLE 8:** Frequency of amino acid substitutions detected in the QRDR, including GyrA, GyrB, ParC, and ParE in fluoroquinolone-resistant MRSA and MSSA clinical isolates from blood culture^*a*^^,*c*^

GyrA				ParC				ParE			
Amino acid substitutions	Total *n* (%)	MRSA *n* (%)	MSSA *n* (%)	Amino acid substitutions	Total *n* (%)	MRSA *n* (%)	MSSA *n* (%)	Amino acid substitutions	Total *n* (%)	MRSA *n* (%)	MSSA *n* (%)
S84L	59 (94)	57 (95)	2 (67)	S80F	53 (84)	51 (81)	2 (67)	P451S	16 (25)	16 (27)	0 (0)
S85P	14 (22)	13 (22)	1 (33)	S80Y	8 (13)	8 (13)	0 (0)	N402T	7 (11)	7 (12)	0 (0)
M121C	8 (13)	8 (13)	0 (0)	E84K	8 (13)	8 (13)	0 (0)	L440F	1 (2)	1 (2)	0 (0)
I175M	7 (11)	7 (12)	0 (0)	E84G	4 (6)	4 (6)	0 (0)	D432V	1 (2)	1 (2)	0 (0)
N182I	6 (11)	6 (10)	0 (0)	I195M	1 (2)	1 (2)	0 (0)	ND^*b*^	41 (65)	38 (63)	3 (100)
I131S	5 (8)	5 (8)	0 (0)	N203I	1 (2)	1 (2)	0 (0)				
L188*	4 (6)	4 (7)	0 (0)	P153A	1 (2)	1	0 (0)				
E88K	3 (5)	3 (5)	0 (0)	Q204H	1 (2)	1 (2)	0 (0)				
A176G	2 (3)	2 (3)	0 (0)	R20D	1 (2)	1 (2)	0 (0)				
L167S	2 (3)	2 (3)	0 (0)	ND	2 (2)	1 (2)	1 (33)				
D151*	1 (2)	1 (2)	0 (0)								
D151M	1 (2)	1(2)	0 (0)								
L188Q	1(2)	1 (2)	0 (0)								
M210E	1 (2)	1 (2)	0 (0)								
ND	3 (5)	2 (3)	1 (33)								

^*a*^ "*” indicates stop codon.

^*b*^ ND, not detected.

^*c*^ Empty cells indicate that the corresponding amino acid substitution was not observed for that gene, as substitution types vary among GyrA, ParC, and ParE.

**TABLE 9:** MIC levels of fluoroquinolone-resistant MRSA toward ciprofloxacin and levofloxacin

Strains	Ciprofloxacin (μg/mL)	Levofloxacin (μg/mL)	Strains	Ciprofloxacin (μg/mL)	Levofloxacin (μg/mL)
1	128	7	31	64	6
2	32	5	32	16	4
3	128	7	33	64	6
4	32	5	34	64	6
5	128	7	35	256	8
6	16	4	36	16	4
7	16	4	37	64	6
8	512	9	38	256	8
9	512	9	39	128	7
10	64	6	40	64	6
11	128	7	41	32	5
12	512	9	42	16	4
13	16	4	43	64	6
14	64	6	44	2	1
15	16	4	45	256	8
16	64	6	46	128	7
17	128	7	47	64	6
18	64	6	48	16	4
19	128	7	49	32	5
20	16	4	50	256	8
21	128	7	51	64	6
22	128	7	52	128	7
23	128	7	53	256	8
24	128	7	54	32	5
25	512	9	55	1,024	10
26	16	4	56	256	8
27	32	5	57	1,024	10
28	64	6	58	512	9
29	64	6	59	256	8
30	128	7	60	32	5

**TABLE 10:** MIC levels of fluoroquinolone-resistant MSSA toward ciprofloxacin and levofloxacin

Strains	Ciprofloxacin (μg/mL)	Levofloxacin (μg/mL)
1	256	8
2	1	0
3	256	8

### Correlation analysis

**Fig 5:**
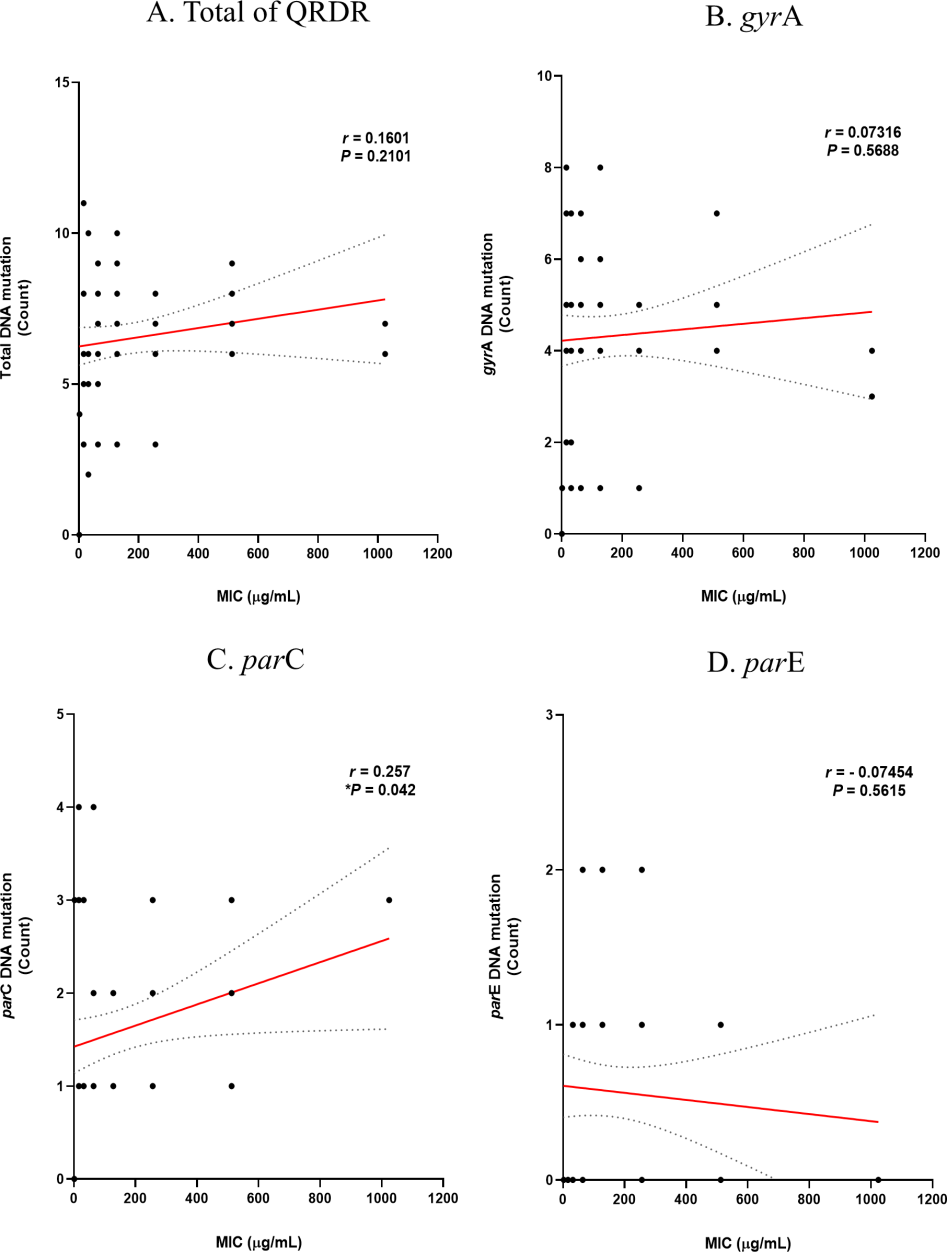
Correlation of DNA mutations in QRDR with MIC levels of ciprofloxacin in MRSA and MSSA. (**A**) Total of QRDR, (**B**) *gyr*A, (**C**) *par*C, and (**D**) *par*E.

**Fig 6:**
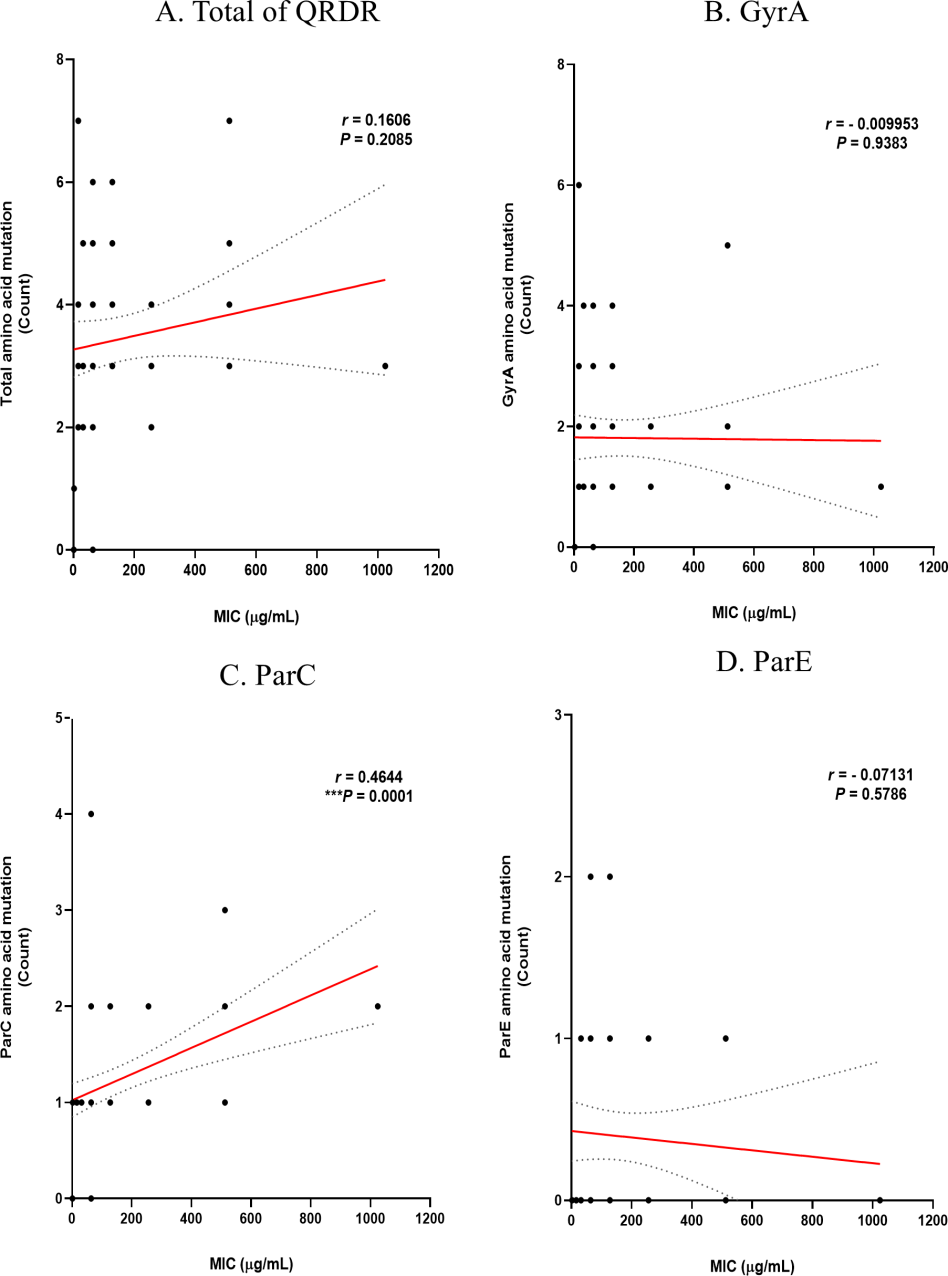
Correlation of amino acid substitutions in QRDR with MIC levels of ciprofloxacin in MRSA and MSSA. (**A**) Total of QRDR, (**B**) GyrA, (**C**) ParC, and (**D**) ParE.

**Fig 7:**
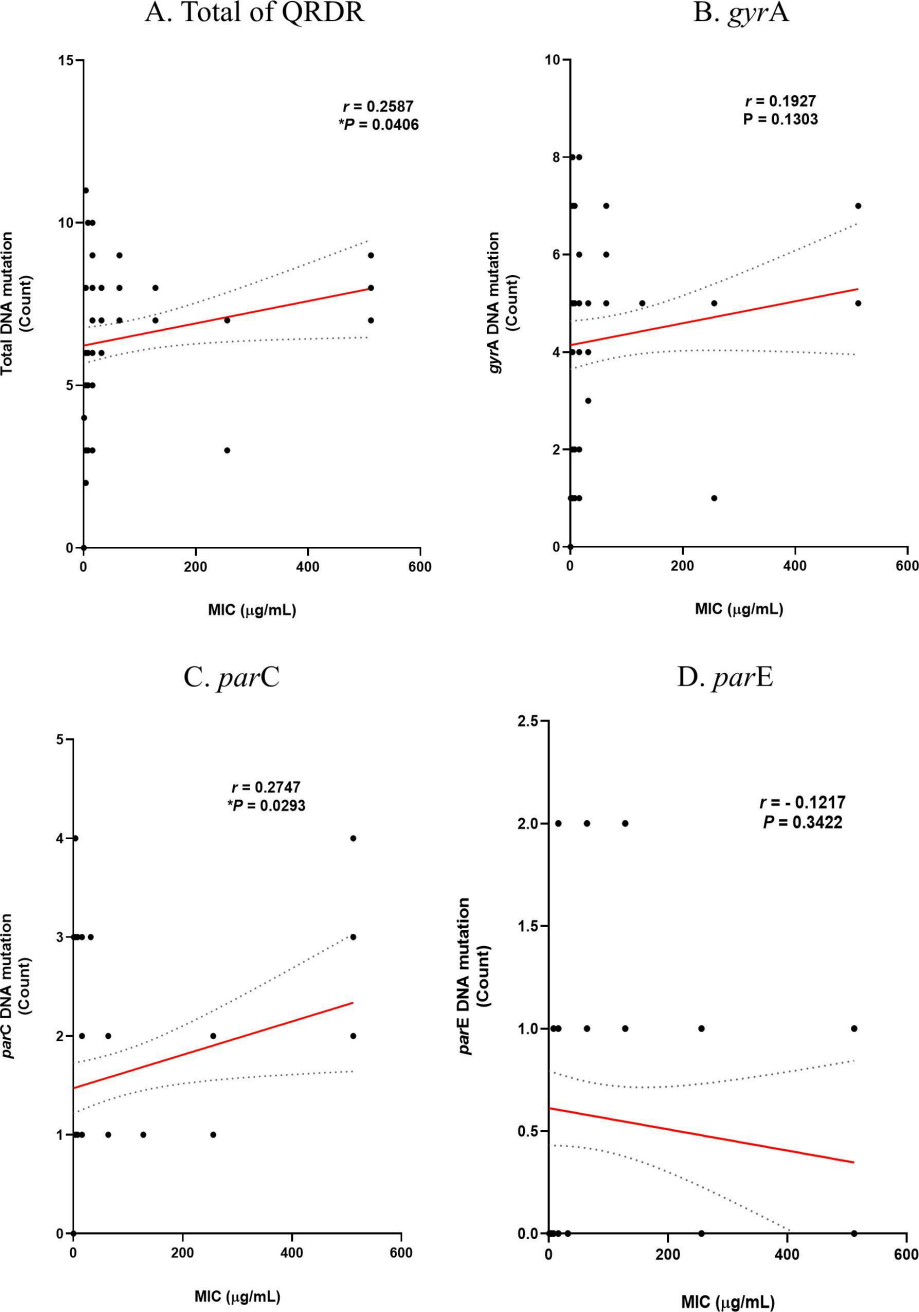
Correlation of DNA mutations in QRDR with MIC levels of levofloxacin in MRSA and MSSA. (**A**) Total of QRDR, (**B**) *gyr*A, (**C**) *par*C, and (**D**) *par*E.

**Fig 8:**
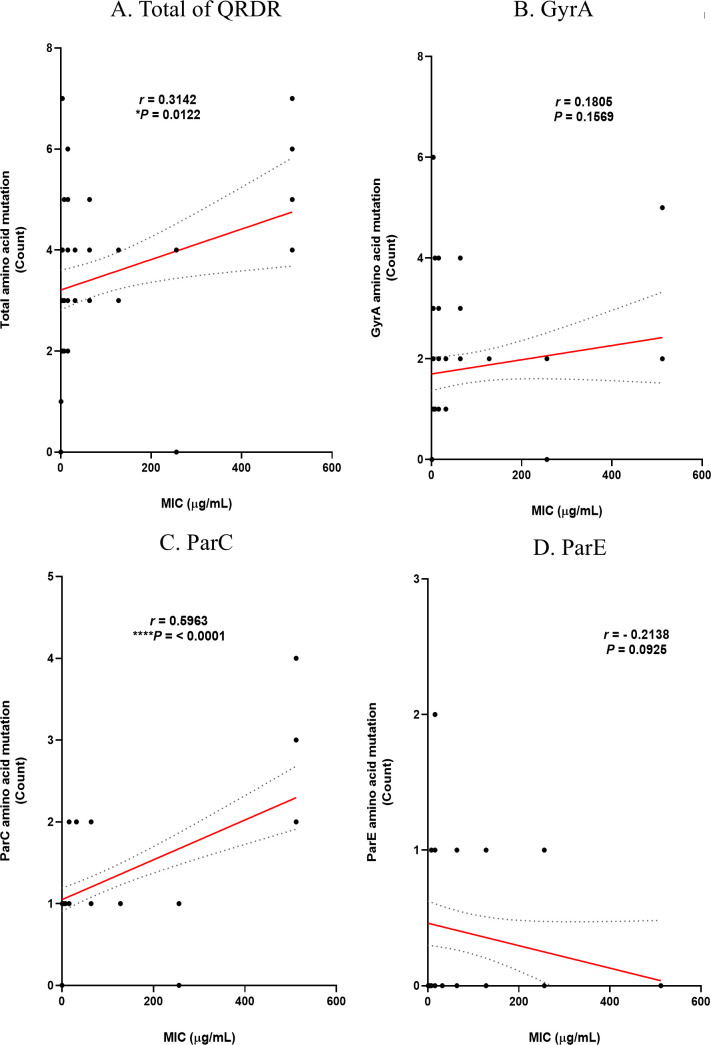
Correlation of amino acid substitutions in QRDR with MIC levels of levofloxacin in MRSA and MSSA. (**A**) Total of QRDR, (**B**) GyrA, (**C**) ParC, and (**D**) ParE.

Based on the previous analysis results, the correlation between the number of mutations and the MIC levels of the two antibiotics was analyzed using Prism. The correlation between the MIC level of ciprofloxacin and DNA mutation is shown in Fig. 5. The correlation *P*-values for mutations and MIC levels in the *gyr*A and *par*E genes were 0.5688 and 0.5615, respectively, resulting in insignificant results. However, the correlation in *par*C was significant (*P = 0.042). The correlation between MIC levels of ciprofloxacin and substitution in amino acids is shown in Fig. 6. As with the DNA level, the correlation *P*-values for substitutions and MIC levels in GyrA and ParE were 0.9383 and 0.5786, respectively, resulting in insignificant results, while the correlation in ParC was significant (***P = 0.0001). These findings suggest that the higher the degree of substitution in ParC, which showed the same correlation at both the DNA and amino acid levels, the higher the ciprofloxacin MIC. The correlation between MIC levels in levofloxacin and DNA mutation is shown in Fig. 7. The correlation *P*-values for mutations and MIC levels in the *gyr*A and *par*E genes were 0.1303 and 0.3422, respectively, resulting in insignificant results, and the correlation in the *par*C gene was significant (*P = 0.0293). The correlation between MIC levels of levofloxacin and substitution in amino acids is shown in Fig. 8. Similar to the DNA level, the correlation *P*-values for substitutions and MIC levels in GyrA and ParE were 0.1569 and 0.0925, respectively, resulting in insignificant results, and the correlation in ParC was significant (****P < 0.0001). These analysis results indicate that substitutions at the ParC site, which are statistically significant for both antibiotics, likely affect the MIC.

In conclusion, it can be inferred that the higher the degree of amino acid substitution at the ParC site among the four subunits of QRDR that determine resistance to fluoroquinolone antibiotics, the higher the MIC level.

### Pattern of amino acid substitutions

Through the correlation analysis of the number of mutations with the MIC, it was found that the substitutions at the ParC in the QRDR affected the MIC. Therefore, we analyzed which substitutions at the amino acid level, expressed as real proteins in ParC, affect the MIC.

We identified the MIC levels and the number of samples for each combination of amino acid substitutions in ParC (Table 11). The group with only the S80F substitution was designated as Group A, the group with S80F and E84K substitutions was designated as Group B, the group with only S80Y substitution was designated as Group C, the group with S80Y and E84G substitutions was designated as Group D, the group with R20D and S80F substitutions was designated as Group E, the group with S80F, E84K, and I195M substitutions was designated as Group F, the group with S80F, E84K, N203I, and Q204H substitutions was designated as Group G, the group with S80F and P153A substitutions was designated as Group H, and Group I was designated as a group that did not show any mutations.

**TABLE 11:** Pattern of ParC amino acid substitutions detected in fluoroquinolone-resistant MRSA and MSSA clinical isolates from blood culture

Group	Pattern of amino acid substitutions in ParC	Total *n* = 63 (100)	MRSA *n* = 63 (100)	MSSA *n* = 3 (100)	MIC level (μg/mL)	
					Ciprofloxacin min-max (median)	Levofloxacin min-max (median)
A	S80F	43 (68)	41 (68)	2 (67)	2–512 (64)	1–256 (16)
B	S80F + E84K	6 (10)	6 (10)	0 (0)	64–512 (384)	16–512 (512)
C	S80Y	4 (6)	4 (7)	0 (0)	16–256 (48)	4–16 (10)
D	S80Y + E84G	4 (6)	4 (7)	0 (0)	256–1,024 (640)	32 (32)
E	R20D + S80F	1 (2)	1 (2)	0 (0)	128 (128)	64 (64)
F	S80F + E84K + I195M	1 (2)	1 (2)	0 (0)	512 (512)	512 (512)
G	S80F + E84K + N203I + Q204H	1 (2)	1 (2)	0 (0)	64 (64)	512 (512)
H	S80F + P153A	1 (2)	1 (2)	0 (0)	128 (128)	16 (16)
I	ND^*a*^	2 (3)	1 (2)	1 (33)	1–64 (32.5)	0.5–256 (128.25)
	Total	63 (100)	60 (100)	3 (100)		

^*a*^ ND, not detected.

Among the 63 *S*. *aureus* strains isolated from patients with sepsis, the most common substitutions combination in ParC was found in Group A (68%). All groups commonly had substitution at codon position 80 (Table 11).

In the case of Group I, which included one MRSA and one MSSA sample, no substitution appeared in ParC, but these samples were resistant to fluoroquinolone drugs. No mutations were found in these samples for the *gyr*A, *gyr*B, or *par*E subunits similar to the *par*C subunit.

Additionally, we compared the MICs of three groups in ParC: group with the substitution at position serine 80 (S80) only (Groups A and C), group with substitutions at both S80 and glutamic acid 84 (E84) simultaneously (Groups B, D, F, and G), and the other group with other substitutions except for the substitution at position E84 (Groups E, H, and I).

**Fig 9:**
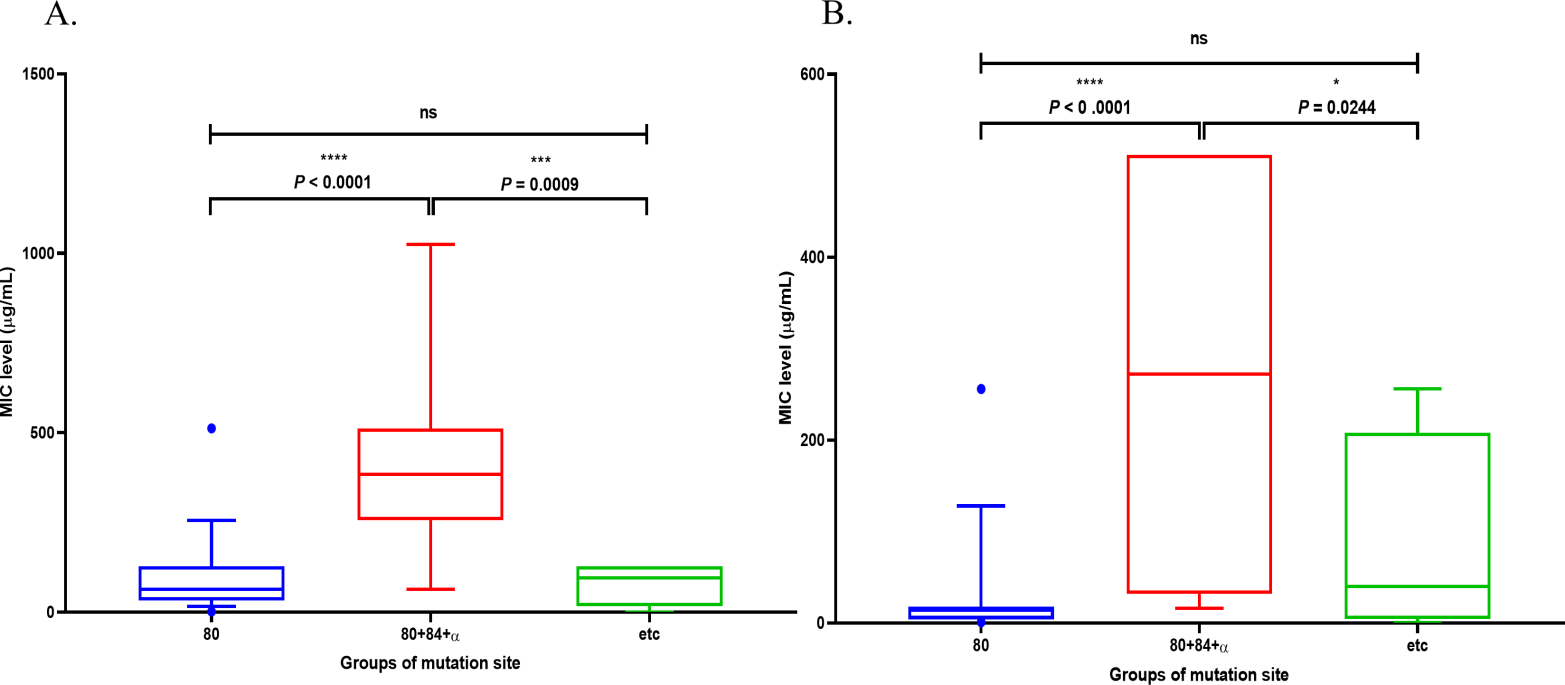
Comparison of fluoroquinolone MIC levels according to substitution site groups. (**A**) Ciprofloxacin, (**B**) Levofloxacin. Groups of mutation site: 80, group with the substitution at position serine 80 only; 80 + 84 + α, group with substitution at both serine 80 and glutamic acid 84 simultaneously; etc., the other group with other substitutions except for the substitution at position glutamic acid 84.

When comparing the ciprofloxacin MIC levels according to the groups of substitution sites, the group with substitutions at both S80 and E84 had significantly higher MIC values compared to the group with the S80 substitution alone and the group with other substitutions (****P < 0.0001, ***P = 0.0009). Similarly, when comparing levofloxacin MIC levels according to the groups of substitution sites, the group with substitutions at both S80 and E84 had significantly higher MIC values compared to the group with the S80 substitution alone and the group with other substitutions (****P < 0.0001, *P = 0.0244) (Fig. 9).

## DISCUSSION

AMR is a global health problem that is expected to cause many deaths in the future (21). Among several antibiotic-resistant bacteria, *S. aureus* is a major cause of HAIs, especially MRSA, a representative pathogen with a consistently high mortality rate (22). *S. aureus* is resistant to several antibiotics, including methicillin, and fluoroquinolone resistance is particularly difficult to treat. The frequency of fluoroquinolone-resistant MRSA is particularly high (17). As antibiotic consumption increases worldwide, studies on antibiotic resistance mechanisms are necessary because the crisis of antibiotic resistance must be overcome.

Therefore, in the present study, we analyzed mutations in the QRDR, including DNA gyrase and topoisomerase IV subunit sites known to determine resistance to quinolone drugs, using *S. aureus* clinical isolate that is resistant to fluoroquinolone in patients with sepsis in the Republic of Korea. In addition, the MIC test for ciprofloxacin and levofloxacin was conducted using the broth microdilution method. Then, we analyzed the correlation between the degree of mutation in QRDR and the MIC level of ciprofloxacin and levofloxacin. Results for the correlation of the number of DNA mutations and amino acid substitutions in QRDR with MIC levels of ciprofloxacin and levofloxacin show that the correlation in *par*C was significant among *gyr*A, *gyr*B, *par*C, and *par*E in QRDR involved in resistance to fluoroquinolone antibiotics. Namely, it suggests that the higher the degree of amino acid substitution at the ParC site among the four subunits of QRDR that determine resistance to fluoroquinolone antibiotics, the higher the MIC level. Furthermore, in ParC, when the substitutions at E84K and E84G occurred with the S80F and S80Y substitutions, the MIC level was markedly increased. Additionally, the C to T mutation appeared most frequently among DNA base mutations in all QRDR sites of *S. aureus*. This result was consistent with previous studies showing that DNA deamination in *Escherichia coli* frequently causes these mutations (23). This suggests that the QRDR site of fluoroquinolone-resistant *S. aureus* is also likely to be influenced by mechanisms related to the deamination of DNA.

In conclusion, the results of the present study indicate that the mutation level of *par*C in QRDR might affect the resistance to fluoroquinolone drugs such as ciprofloxacin and levofloxacin. Additionally, the simultaneous appearance of substitutions at E84 with S80 might affect the high resistance of fluoroquinolone drugs such as ciprofloxacin and levofloxacin. This result agrees with a previous study showing that the simultaneous appearance of substitutions at E84 with S80 affects the high resistance to norfloxacin in fluoroquinolones (10). Also, these results agree with previous studies that have shown high ciprofloxacin resistance in *S. aureus* with double substitutions, where S80 and either E84 or A48 substitutions co-occurred in ParC (24, 25). However, there have been studies showing that these double substitutions were not associated with high levofloxacin resistance (24). In contrast, we found that these double substitutions were also associated with high levofloxacin resistance in *S. aureus* from the Republic of Korea in this study (Fig. 9). Similarly, there is also a result showing that delafloxacin-resistant *S. aureus* isolates exhibited high resistance when the 84th substitution occurred in ParC (26). Additionally, in ParC, all isolates except non-mutation isolates had either S80Y or S80F substitutions. These findings were consistent with previous studies showing that serine substitution occurs most frequently in QRDR sites, and it is expected that serine substitution is the main cause of quinolone resistance. It will be easier to identify resistant bacteria if a method to quickly and easily detect it is developed (13). Furthermore, silent base mutation isolates also existed. Notably, one isolate in Group I exhibited no mutations in any of the QRDR (*gyr*A, *gyr*B, *par*C, and *par*E), despite showing resistance. This suggests that mechanisms other than QRDR mutations—such as efflux pump overexpression or plasmid-mediated resistance—may be involved in conferring fluoroquinolone resistance in certain strains. Therefore, further studies are required to confirm the corresponding resistant mechanisms, such as the efflux pump genes, including *nor*A, *nor*B, and *nor*C. In addition, our study also identified several less commonly reported substitutions, including E84G and combinations such as S80F + E84G, as well as rare variants like R20D, I195M, Q204H, and P153A. To our knowledge, these latter mutations have not been frequently documented in existing literature, suggesting the possibility of regional or strain-specific mutation patterns. Further phylogenetic or comparative genomic analyses may be needed to determine whether such patterns are lineage-dependent.

In summary, the present study confirms that the most frequent DNA base mutation in the QRDR of fluoroquinolone-resistant *S. aureus* was C to T. Additionally, the degree of mutation in the *par*C region of QRDR affects the MIC level. Furthermore, the E84 position is an important factor in the treatment and resistance management of *S. aureus* isolated from hospitals in the Republic of Korea that exhibit high resistance to fluoroquinolone agents such as ciprofloxacin and levofloxacin.

Results of these studies on the mechanisms of antibiotic resistance are expected to contribute to the development of effective antibiotic treatments, infection prevention and control, and improved diagnostic methods. They will also play an important role in creating an environment that reduces the indiscriminate use of antibiotics.
